# Diagnostic and Prognostic Implications of Ultra-Low-Profile INCRAFT and Ovation Endografts: Long-Term Follow-Up in a Single-Center Experience

**DOI:** 10.3390/diagnostics16081187

**Published:** 2026-04-16

**Authors:** Fabio Massimo Oddi, Rosario Micali, Andrea Cuoghi, Grazia Granata, Manuel Romano, Federico Francisco Pennetta, Mauro Fresilli, Andrea Ascoli Marchetti, Eugenio Martelli

**Affiliations:** Vascular Surgery Unit, Department of Biomedicine and Prevention, University of Rome “Tor Vergata”, 00133 Rome, Italy

**Keywords:** abdominal aortic aneurysm, endovascular aneurysm repair, ultra-low-profile endograft, INCRAFT, Ovation

## Abstract

**Background/Objectives**: Ultra-low-profile (ULP) endografts have expanded the applicability of endovascular aneurysm repair (EVAR) in patients with challenging aortoiliac anatomy and narrow access vessels. However, direct long-term comparisons between different ULP devices remain limited. This study aimed to compare mid- to long-term outcomes of the INCRAFT and Ovation endografts in a single-center experience. **Methods**: This retrospective single-center study included 102 patients (45 Ovation, 57 INCRAFT) with a median follow-up exceeding 60 months. We retrospectively analyzed 102 consecutive patients undergoing elective EVAR with ULP devices between January 2011 and December 2019. Forty-five patients were treated with Ovation and 57 with INCRAFT. The primary endpoint was technical success. Secondary endpoints included survival, reintervention, endoleak, and device-related complications. Statistical comparisons were performed using Student’s *t*-test and Fisher’s exact test. **Results**: Primary technical success was achieved in all cases. The Ovation group exhibited more complex proximal neck anatomy, including greater thrombus involvement (47.4% vs. 12.7%, *p* < 0.001). Post-implantation syndrome occurred more frequently with INCRAFT (14% vs. 0%, *p* = 0.009). No significant differences were observed in endoleak, major adverse events, or total reintervention. Long-term mortality was higher in the Ovation group (37.8% vs. 15.8%, *p* = 0.01), although deaths were not aneurysm-related. Median follow-up exceeded 60 months in both groups. **Conclusions**: Both ULP endografts demonstrated favorable long-term outcomes within the limitations of a non-randomized, anatomically heterogeneous cohort.

## 1. Introduction

Despite continuous advancement in endovascular technologies, challenging aortic anatomy, including hostile proximal aortic necks, anatomically smaller iliac arteries, iliac occlusive disease, iliac artery stenosis and difficult access vessels still limit the applicability of endovascular aortic aneurysm exclusion (EVAR), with conventional devices, in patients affected by abdominal aortic aneurysm (AAA) [1,2].

The advent of ultra-low-profile (ULP) endografts with a 14 F outer diameter has allowed the feasibility of EVAR of infrarenal AAA in patients who have previously been excluded because of challenging aortic anatomies and small and tortuous access vessels [3]. Accurate preoperative anatomical assessment and postoperative imaging surveillance are essential for diagnostic evaluation and prognostic stratification in these patients. Preoperative imaging plays a fundamental role in the diagnostic evaluation of abdominal aortic aneurysm morphology and access vessel characteristics. Computed tomography angiography (CTA) enables precise preoperative assessment of aortic neck anatomy, thrombus burden, and iliac characteristics, guiding endograft selection. Postoperative imaging surveillance provides prognostic information on device durability and complications, including endoleak and graft limb occlusion.

Among the devices now available, the ultra-low-profile endografts Ovation (Endologix, Santa Rosa, CA, USA) and INCRAFT (Cordis Corp., Milpitas, CA, USA) have been shown to be safe and effective in patients with heavily calcified external and common iliac axes, or with aortoiliac occlusive disease [4,5,6,7,8,9,10].

However, direct comparisons between ultra-low-profile devices in long-term, real-world single-center experiences remain limited.

The purpose of this article is to compare the mid-term follow-up of these two endoprostheses.

## 2. Materials and Methods

This is a single-center, retrospective, observational study using data extracted from medical records. Informed consent was obtained from all patients. The study was conducted in accordance with the Declaration of Helsinki. According to institutional policy, formal ethical approval was waived due to the retrospective nature of the study. All patients submitted to elective EVAR in our institute using the Cordis Incraft AAA Stent Graft System and the Trivascular Ovation endografts from January 2011 to December 2019 were enrolled. In order to compare cases with similar aneurysm location and graft burden, all urgent EVAR patients with a dissecting or ruptured aneurysm and patients with a history of connective tissue disease were excluded. Inclusion criteria were: patients undergoing elective EVAR for infrarenal abdominal aortic aneurysm using ultra-low-profile endografts. Exclusion criteria included: urgent procedures, ruptured or dissecting aneurysms, connective tissue disorders, and incomplete follow-up data.

The selection of the EVAR endograft was based on the anatomical characteristics of the aortic neck and iliac arteries, the configuration of the aneurysm sac, the presence of thrombus or calcifications, and the number of patent side branches arising from the aneurysmal sac. The final choice also depended on the surgeon’s preference and the availability of suitable devices at the time of the procedure. Although overall aneurysm characteristics were comparable, some anatomical differences were observed between groups. We specifically opted for the use of a ULP endograft in case of small and tortuous iliac vessels, iliac occlusive disease or iliac artery stenosis. In particular, a ULP device was chosen when the external iliac-femoral arteries were smaller than 7 mm in caliber. All patients underwent preoperative CTA scans to plan the endovascular treatment based on the aortoiliac morphology. All measurements of the aneurysm were performed using dedicated software (OsiriX MD (v4.1.2); Pixmeo Labs, Geneva, Switzerland; Aquarius Software (v4.4), TeraRecon, Inc., San Mateo, CA, USA). All patients received prophylactic antibiotic treatment (Cefazolin 2 g) before the procedure and 5.000 International Units of unfractionated heparin before insertion of the stent graft deployment system. Local or general anesthesia was selected by the anesthesiologist team. All patients received antiplatelet therapy immediately after the endovascular procedure and continued throughout the follow-up. The primary endpoint was technical success. Secondary endpoints included survival, reintervention rate, and device-related complications.

Procedural data included fluoroscopy time, operation time, amount of procedural contrast medium, blood loss, and any complications. All patients were on standard cardiovascular medical therapy preoperatively according to their comorbidities. Postoperatively, all patients received single antiplatelet therapy (aspirin), unless already on chronic anticoagulation, in which case therapy was maintained. Follow-up data were analyzed to evaluate technical success, survival, complications, and device-related events, both at 30 days, in the mid-term and long-term. Mid-term follow-up was defined as 12–36 months, while long-term follow-up was defined as greater than 36 months. Technical success was defined as successful access, delivery and implant of the endograft with absence of immediate surgical conversion, mortality, type I or III endoleak, or graft limb occlusion. Endoleaks were classified according to standard reporting criteria (types I–V) and detected using CTA or duplex ultrasound during follow-up. Reinterventions were defined as any secondary procedure performed to treat device-related complications or aneurysm-related issues. Postoperative surveillance protocol included a duplex ultrasonography (DUS) scan at discharge, at 1, 6, and 12 months, and annually thereafter. A CTA was performed at 1 month and in case of a non-diagnostic DUS scan or if either graft thrombosis or endoleak was suspected at the DUS scan. Therefore, in summary, CTA was preferred in the early postoperative period and in cases of suspected complications, while duplex ultrasound was routinely used during long-term surveillance.

### Statistical Analysis

All patients were divided into 2 groups: Incraft and Ovation. Mean and median values were calculated for numeric variables. Categorical variables, proportions and significance tests on the 5% significance level were performed (considering a *p* < 0.05 of significance). Categorical variables were reported as a percentage (%), and continuous variables were expressed as mean ± SD. A nonparametric significance test between the two groups was used for qualitative variables, while Student’s *t*-test was used for quantitative variables. To quantify the degree of baseline imbalance between groups, standardized mean differences (SMD) were calculated for all baseline variables. An SMD > 0.2 was considered indicative of moderate imbalance, and an SMD > 0.5 of substantial imbalance. Survival and freedom from reintervention were additionally analyzed using Kaplan–Meier curves, and differences between groups were assessed using the log-rank test. A two-sided *p*-value < 0.05 was considered statistically significant. Analyses were performed using computer software packages (SPSS-26.0, SPSS Inc. Chicago, IL, USA).

## 3. Results

Between January 2011 and December 2019, a total of 102 patients were enrolled. Among these, 45 (44.1%) underwent EVAR with the Trivascular Ovation device and 57 (55.9%) with the Cordis INCRAFT device.

The mean age was 74.1 ± 7.8 for the Ovation group and 74.3 ± 7.2 for the Incraft group, and most were male (86% in the Ovation group and 91% in the Incraft group). The baseline demographic characteristics of the studied population are listed in Table 1. The majority of cardiovascular risk factors were equally distributed among the two groups, except for COPD (80% in the Ovation group and 49.1% in the Incraft group) (Table 1).

The anatomical features are shown in Table 2. The two groups were quite different in terms of aorto-iliac anatomy. The Ovation group had a greater percentage of the circumference covered by thrombus in the proximal aortic neck (47.4% vs. 12.7%) (Table 2).

Standardized mean differences confirmed substantial baseline imbalance for aortic bifurcation diameter (SMD = 0.91), % circumference covered by thrombus of aortic neck (SMD = 2.29), and CIA diameter (SMD = 0.55), reflecting the anatomy-driven nature of device selection in this cohort.

The intraoperative details are shown in Table 3. Bilateral percutaneous access was performed in 100% of cases for Ovation and in 91.6% for Incraft. Primary intraoperative technical success was achieved in all cases, with no intraoperative complications. The bilateral femoral percutaneous approach is the preferred technique without major complications and with good results (Table 3).

The outcome (early complications and long-term results) is shown in Table 4. Reintervention occurred in six patients in the Incraft group (mostly embolization and/or stenting, and one thromboaspiration of the popliteal artery and tibial vessels in a patient within 30 days from the operation) and in one patient in the Ovation group (explant of the stent-graft). The explanation was performed due to Endoleak type I with distal migration of the stent-graft. No significant differences were observed between the two groups in terms of endoleak. Post-implantation syndrome was noted in the Incraft group (14% vs. 0%, *p* = 0.009). At follow-up, eighteen patients died in the Ovation group and thirteen in the Incraft group, not AAA-related (Table 4).

**Figure 1:**
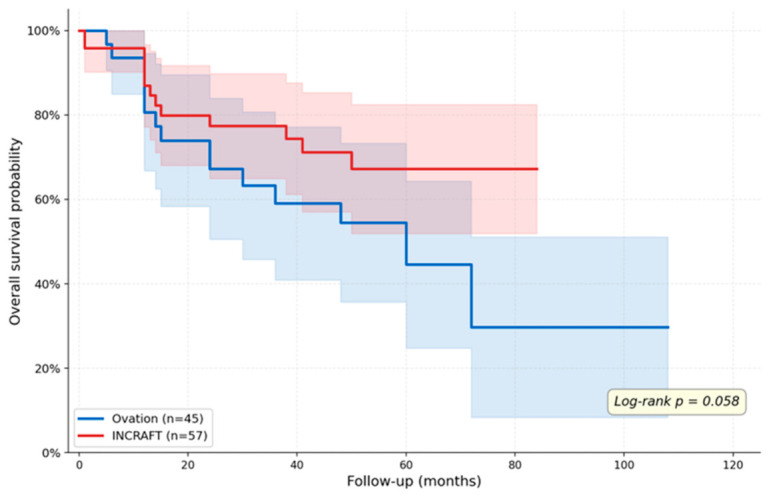
Kaplan–Meier survival curves comparing overall survival between patients treated with INCRAFT and Ovation endografts. Kaplan–Meier survival analysis showed a trend toward lower overall survival in the Ovation group compared to the INCRAFT group; however, this difference did not reach statistical significance (log-rank *p* = 0.058).

**Figure 2:**
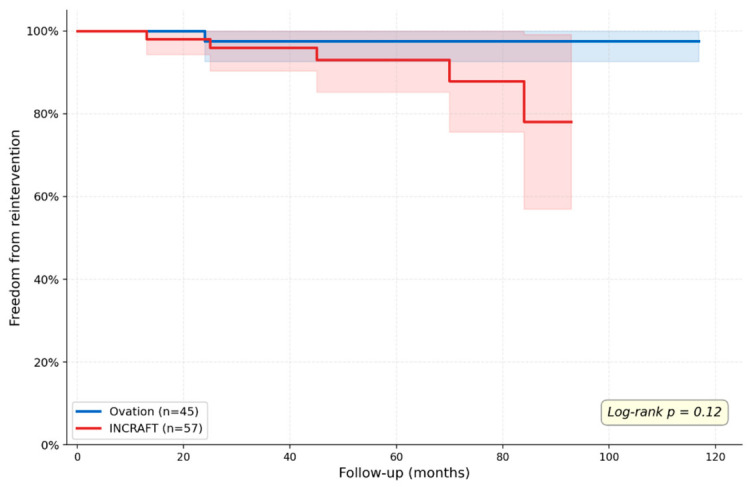
Kaplan–Meier curves showing freedom from reintervention in the Ovation and INCRAFT groups over a median follow-up exceeding 60 months. No statistically significant difference was observed between the two groups (log-rank *p* = 0.12).

Kaplan–Meier survival analysis did not show statistically significant differences in overall survival between the two groups (log-rank *p* = 0.058) (Figure 1). Similarly, freedom from reintervention was comparable (log-rank *p* = 0.12) (Figure 2). The median follow-up time was 64.3 months (up to 117 months) for the Ovation group, 62.4 months (up to 93 months) for the Incraft group.

## 4. Discussion

The introduction of ULP endografts with an outer diameter of 14 F has enabled the feasibility of EVAR of infrarenal AAA in patients previously excluded due to difficult aortic anatomy and small and tortuous access vessels, eliminating the need for preparation vessels (femoral exposure, iliac dottering, iliac conduit, endoconduit) and reducing main and groin access complications [11,12,13].

In particular, the presence of narrow access vessels (small iliac arteries, iliac occlusive disease, iliac artery stenosis and difficult access vessels) continues to limit the applicability of endovascular aortic aneurysm exclusion (EVAR), and access-related complications occur in up to 17% of cases [10].

Ultra low-profile endografts such as Incraft and Ovation address the need to extend the suitability of EVAR to patients with complex anatomy by eliminating access problems and improving device insertability [14].

CTA is the reference standard for preoperative planning in EVAR, providing a detailed assessment of aneurysm morphology, proximal neck anatomy, thrombus burden, and iliac access vessels. These parameters are critical for device selection, particularly when ultra-low-profile endografts are considered for patients with challenging anatomy. Precise measurement of landing zones and vascular diameters further enables a tailored endovascular strategy, improving procedural safety and feasibility [15,16]. Several studies have demonstrated that systematic imaging surveillance is associated with improved detection of clinically relevant complications and contributes to long-term outcome evaluation in EVAR patients [16,17,18,19].

Our results are in line with previous studies reporting favorable mid- to long-term outcomes for both devices. The safety and efficacy of these two stent-grafts are well described in the literature. Already in 2017, Mazzaccaro D. et al. showed satisfying early and mid results of the two ULP (Incraft and Ovation), describing technical differences among them [3]. The INNOVATION Trial confirmed the efficacy and low-complications rate of Incraft stent-graft through 4 years of follow-up, while Ovation stent-graft was well documented in a short- to mid-term experience, reporting particularly the safety and efficacy of this ULP in patients off IFU (Instructions for Use) with hostile neck anatomy [20]. On the other hand, the long-term outcomes of ULP have been shown by De Donato G. et al. in an 8-year follow-up, reporting overall survival and AAA-related death in line with those reported for traditional devices [11]. Most previous studies are multicenter registries, whereas our monocentric experience allows consistent surgical technique and standardized follow-up [21,22]. Importantly, the overall survival and reintervention rates observed in our cohort are comparable to those reported for standard EVAR devices, suggesting that ultra-low-profile systems do not compromise long-term outcomes despite being used in more complex anatomical settings. In our experience at a median follow-up of over 60 months, both devices showed favorable durability profiles in their respective anatomical settings with low rates of endoleak, reintervention, mortality and major adverse events, in line with data in the literature.

A ULP device was chosen when the external iliac-femoral arteries had a caliber smaller than 7 mm, and when the diameter of the aortic bifurcation was less than 16 mm, we chose to use the INCRAFT.

The Cordis Incraft stent graft is a trimodular, self-expanding nitinol hypotube covered by a low porosity sutureless Dacron graft with a main body for the aortic bifurcation, suprarenal fixation and two iliac limb endoprostheses. It has been designed and engineered with the undeniable advantage of combining navigability in difficult access with adaptability to most anatomies, ensuring durability, adaptability and sealing. The delivery system has an outer diameter of 14 Fr for the 22- to 30 mm main body and 16 Fr for the 34 mm main body, with an integrated and braided sheath introducer [23,24].

Trivascular Ovation is a trimodular device. Sealing is achieved by a ring filled with a radiopaque viscose polymer under a pressure of 1 Atm 13 mm below the renal arteries (effective sealing) and a suprarenal 35 mm free-flow stent (active fixation): sealing and fixation are maintained separately. With the Ovation endograft platform, the complete absence of a metallic endoframe maintains a lower profile while achieving a good proximal seal and likely good durability over time. It is delivered via a flexible, hydrophilic-coated catheter with an outer diameter of 14 Fr (OD) for the 20- to 29 mm main body and 15 Fr for the 34 mm main body [25]. The differences in sealing mechanisms between the two devices may partially explain their behavior in different anatomical settings. The Ovation platform relies on a polymer-based sealing ring, which allows effective sealing even in hostile neck anatomy with limited reliance on radial force. In contrast, the INCRAFT device achieves sealing through radial force exerted by the nitinol stent structure, providing greater columnar support, which may be advantageous in narrow aortic bifurcations. These biomechanical differences may influence device selection and long-term performance, particularly in anatomically challenging cases.

The main finding of this study is that favorable mid- to long-term outcomes can be achieved with ultra-low-profile endografts in anatomically selected patients. Our study demonstrates that elective treatment of AAA with a low-profile stent graft is safe and effective, with acceptable durability outcomes as shown by long-term technical success of 88% for Ovation and 92% for Incraft. In particular, although the Ovation group presented more challenging proximal neck characteristics, including a higher thrombus burden, no significant differences were observed in terms of endoleak, reintervention, or device-related complications.

Post-implantation syndrome was noted in the Incraft group (14% vs. 0%, *p* = 0.009), which was attributed to the Dacron graft material, according to the Tor Vergata group study [26]. This finding is likely related to the polyester (Dacron) graft material, which has been associated with a higher inflammatory response compared to other materials. Although long-term mortality was higher in the Ovation group, deaths were not aneurysm-related and likely reflect differences in baseline clinical risk and comorbidities rather than device-related factors. Experimental studies have demonstrated that growth factors such as PDGF and bFGF play a key role in vascular healing and neointimal hyperplasia following graft implantation, potentially influencing long-term device integration and performance [27].

The major limitation of this study is its retrospective, non-randomized design, which resulted in significant baseline differences between groups—particularly in anatomical characteristics that directly drove device selection. Standardized mean differences confirmed substantial imbalance in aortic bifurcation diameter, CIA diameter, and COPD prevalence. As a result, unadjusted comparisons between groups may reflect underlying patient and anatomical differences rather than intrinsic device performance. Multivariable adjustment was not performed given the limited sample size relative to the number of potential confounders, which would have risked model overfitting. These findings should therefore be interpreted as exploratory and hypothesis-generating rather than as evidence of device equivalence or comparative effectiveness. Further prospective studies with larger cohorts and adjusted analyses are needed.

The present study, despite its retrospective and monocentric design, represents one of the longest single-center comparative experiences reported to date for these two ultra-low-profile devices, with a median follow-up exceeding 60 months in both groups. The long inclusion period (2011–2019) may also reflect temporal changes in operator experience, device technology, and perioperative management, which could have influenced outcomes. The comparability of mid- to long-term outcomes between INCRAFT and Ovation—in terms of endoleak rate, reintervention, and device-related complications—suggests that the choice between the two devices should be guided primarily by individual anatomical characteristics rather than by expected differences in clinical performance [28,29]. In particular, the Ovation platform may be preferred in patients with more hostile proximal neck anatomy, given its polymer-based sealing mechanism and the documented off-IFU experience reported in the literature, whereas INCRAFT may represent a more appropriate choice in cases of narrow aortic bifurcation, given its superior columnar support and precise iliac limb adjustability [30,31,32]. These findings suggest that device selection in EVAR with ultra-low-profile systems should be primarily guided by anatomical considerations rather than expected differences in long-term performance. The ability of the Ovation platform to achieve effective sealing in hostile neck anatomy, combined with the structural advantages of INCRAFT in narrow aortic bifurcations, supports a tailored approach based on patient-specific vascular morphology.

## 5. Conclusions

Ultra-low-profile endografts represent a reliable and effective option for the endovascular treatment of abdominal aortic aneurysms in patients with challenging aortoiliac anatomy. In this real-world single-center experience, favorable mid- to long-term outcomes were observed with both ULP endografts in anatomically selected patients. However, given the non-randomized design and the substantial baseline imbalance between groups, these findings should be interpreted with caution and considered descriptive and hypothesis-generating rather than evidence of comparative effectiveness or device equivalence. The bilateral femoral percutaneous approach was confirmed to be the preferred technique, with no major access-related complications. These findings support a patient-tailored approach to device selection, driven primarily by anatomical characteristics rather than intrinsic device performance. The main limitations of this study include its retrospective nature and the fact that, in the early part of our experience, Ovation was the only ULP endograft available. Further prospective studies with adjusted analyses are needed to draw more definitive conclusions on comparative device performance and to assess whether ULP endografts maintain their safety profile beyond the current 8-year follow-up experience.

## Figures and Tables

**Table 1:** Baseline demographic characteristics and cardiovascular risk factors of patients treated with ultra-low-profile endografts. Data are reported as mean ± standard deviation (SD) or number (percentage). Statistical comparisons between groups were performed using Student’s *t*-test for continuous variables and Fisher’s exact test for categorical variables. SMD = Standardized Mean Difference. Values > 0.2 indicate moderate imbalance; values > 0.5 indicate substantial imbalance between groups.

Comorbidities	Ovation (*n* = 45)	Incraft (*n* = 57)	*p*	SMD
Male sex, *n* (%)	39	51 (91%)	0.66	0.09
Age (years), mean ± SD	74.1 ± 7.8	74.3 ± 7.2	0.23	0.05
Current or previous smoking	36 (80%)	45 (79.1%)	0.89	0.03
COPD	36 (80%)	28 (49.1%)	0.01	0.64
CAD	14 (31.1%)	21 (36.8%)	0.54	0.16
Hypertension	39 (86.7%)	49 (86%)	0.91	0.04
Dyslipidemia	30 (66.7%)	32 (56.1%)	0.28	0.23
Diabetes	8 (17.8%)	14 (24.6%)	0.40	0.02
Renal Failure	7 (15.6%)	9 (15.8%)	0.97	0.07
Anticoagulant therapy	7 (15.6%)	9 (15.8%)	0.97	0.01

**Table 2:** Preoperative anatomical characteristics of the abdominal aortic aneurysms and access vessels in the Ovation and Incraft groups. Continuous variables are expressed as mean ± SD. *p*-values refer to intergroup comparisons. SMD = Standardized Mean Difference. Values > 0.2 indicate moderate imbalance; values > 0.5 indicate substantial imbalance between groups.

	Ovation (*n* = 45)	Incraft (*n* = 57)	*p*	SMD
**Anatomical data (mm), (mean ± SD)**				
Proximal aortic neck diameter	23.7 ± 2.9	22.9 ± 2.5	0.13	0.16
Proximal aortic neck length	15.4 ± 8.8	20.5 ± 11.7	0.02	0.23
Proximal aortic neck angulation (coronal axis)	36° ± 18°	34.4° ± 14.1°	0.62	0.15
% of circumference covered by thrombus (aortic neck)	47.4	12.7	<0.001	2.29
Aortic bifurcation diameter (mm)	19.9 ± 4.5	24.9 ± 8.9	0.03	0.91
**Access vessel anatomy**				
Mean CIA diameter (mm)	13.05 ± 2.4	14.81 ± 3.6	0.01	0.55
Mean EIA diameter (mm)	6.7 ± 1.5	7.5 ± 1.8	0.02	0.33

**Table 3:** Intraoperative procedural details and technical outcomes. Continuous variables are expressed as mean ± SD and categorical variables as number (percentage). Procedural success was defined as successful endograft deployment without intraoperative complications.

	Ovation (*n* = 45)	Incraft (*n* = 57)	*p*
Percutaneous vascular access	45 (100%)	52 (91.6%)	0.07
Time of operation (min), mean ± SD	98 ± 25	96.5 ± 72.2	0.80
Amount of contrast (mL), mean ± SD	63.7 ± 29.6	54 ± 21.6	0.08
Blood loss (mL), mean ± SD	62.2 ± 22.9	62.1 ± 20.4	0.98
Procedural success	45 (100%)	57 (100%)	-
Accidental hypogastric artery coverage	0	0	-
Intraoperative complications	0	0	-

**Table 4:** Early (30-day) and long-term clinical outcomes following elective EVAR with ultra-low-profile endografts. Continuous variables are expressed as mean ± SD and categorical variables as number (percentage). *p*-values indicate comparisons between the Ovation and Incraft groups.

	Ovation (*n* = 45)	Incraft (*n* = 57)	*p*
**30-day complications**			
Post-implantation syndrome	0	8 (14%)	0.009
Reintervention	0	1 (1.7%)	0.37
Major adverse events	0	0	-
**Long-term results**			
Death	17 (37.8%)	9 (15.8%)	0.01
Major adverse events	5 (11.1%)	6 (10.5%)	0.92
Endoleak	9 (20%)	13 (22.8%)	0.73
Explant	1 (2.2%)	0	0.25
Total reintervention	1 (2.2%)	6 (10.5%)	0.10
Occlusion	0	2 (3.5%)	0.20
Follow-up (months); median (IQR; range)	64.37 ± 30.5	62.47 ± 19.4	0.71

## Data Availability

The raw data supporting the conclusions of this article will be made available by the authors on request.

## Abbreviations

The following abbreviations are used in this manuscript:

EVAR	Endovascular Aneurysm Repair
AAA	Abdominal Aortic Aneurysm
ULP	Ultra low-profile
CTA	Computed Tomography Angiography
DUS	Duplex Ultrasonography
COPD	Chronic Obstructive Pulmonary Disease
IFU	Instructions For Use
OD	Outer diameter
